# Glucocorticoid receptor repression mediated by BRCA1 inactivation in ovarian cancer

**DOI:** 10.1186/1471-2407-14-188

**Published:** 2014-03-14

**Authors:** Yuan-Yuan Fang, Da Li, Chen Cao, Chun-Yan Li, Ting-Ting Li

**Affiliations:** 1 Department of Obstetrics and Gynecology, Shengjing Hospital, China Medical University, Shenyang 110004, China; 2 Department of Pathology, Chinese PLA General Hospital, Beijing 100853, China; 3 Department of Histology and Embryology, Institute of Basic Medical Sciences, Chinese Academy of Medical Sciences, School of Basic Medicine Peking Union Medical College, Beijing 100005, China; 4 Department of Medical Oncology, Shengjing Hospital, China Medical University, Shenyang 110004, China

**Keywords:** BRCA1, BRCA2, Glucocorticoid receptor, Ovarian cancer

## Abstract

**Background:**

BRCA mutations are the main known hereditary factor for ovarian cancer. Notably, emerging evidence indicates that the glucocorticoid receptor (GR) has drawn considerable interest in ovarian cancer development. However, dynamic cross-talk between BRCA1 and GR signaling pathways are poorly understood.

**Methods:**

The regulatory effects of BRCA on GR were assessed in 146 serous ovarian cancer patients (28 pairs of BRCA1-mutated or not, 23 pairs of BRCA2-mutated or not, and 22 pairs with hypermethylated BRCA1 promoter or not). BRCA1 promoter methylation was analyzed by bisulfite sequencing using primers flanking the core promoter region. Expression levels of BRCA1 and GR were assessed by immunohistochemistry and real-time PCR. Regression analysis was used to examine the possible relationship between BRCA1 and GR expression levels. The knockdown and overexpression of BRCA1 were achieved using a lentiviral vector in 293 T cells, SKOV3 ovarian cancer cells, and primary non-mutated and BRCA1-mutated ovarian cancer cells.

**Results:**

GR expression levels were unchanged in non-BRCA1-mutated, non-BRCA2-mutated and BRCA2-mutated ovarian cancer compared to their normal tissues; BRCA1 repression (BRCA1 mutation or BRCA1 promoter hypermethylation) ovarian cancer showed decreased GR levels compared to normal tissue; there was a positive correlation between BRCA1 and GR expression in human ovarian cancer specimens; BRCA1 knockdown was effective at inhibiting GR expression, and overexpression of BRCA1 induces an increase in GR levels in ovarian cancer cells.

**Conclusions:**

These results suggest that GR may be a potential target for BRCA1 in ovarian cancer progression.

## Background

Ovarian cancer is the most lethal gynecological malignancy in women worldwide [1]. To date, although the exact cause of ovarian cancer remains largely unknown, BRCA mutations are the main known hereditary factor [2], and the risk of ovarian cancer conferred by BRCA mutations can be regulated by both genetic and environmental components [3]. Glucocorticoid action in cells is mediated by the glucocorticoid receptor (GR), a member of the superfamily of ligand-inducible transcription factors that exert a variety of physiological functions, such as inflammation, autoimmune diseases, and cancer [4]. Recently, the glucocorticoid system has drawn considerable interest in the field of ovarian cancer therapy, with studies involving, for instance, glucocorticoids-induced chemotherapy resistance in ovarian cancer cells [5,6]; GR may be involved in the pathogenesis of ovarian cancer via the regulation of apoptosis and aberrant cell migration [7]. In addition, emerging evidence has suggested that: (i) both genetic and environmental factors contribute to impaired GR function [8]; (ii) GR inactivation is a hallmark for BRCA1-mutated breast cancer tissues [9]; and (iii) the BRCA1-interacting protein NELF-B participates in GR-mediated gene induction [10]. However, to date, little is known about the effects of BRCA dysfunction on GR in ovarian cancer. Therefore, insights into the complex interrelationship between BRCA and GR might improve our understanding of the basic molecular mechanism of ovarian cancer. For this reason, the present study was undertaken to investigate GR expression after BRCA inactivation events (mutation, promoter methylation, or knockdown), and to provide novel insights into the regulatory mechanism of GR in ovarian cancer progression.

## Methods

### Patients and tissue collection

This study was approved by the Institutional Review Board at China Medical University. Serous ovarian cancer patients were enrolled between 2010 and 2012, and all patients gave informed consent. Fresh tumor samples, adjacent normal ovarian tissues, ascites, and blood samples were obtained at the time of primary surgery before any chemotherapy or radiotherapy (28 pairs of BRCA1-mutated or not, 23 pairs of BRCA2-mutated or not, and 22 pairs with hypermethylated BRCA1 promoter or not). Hematoxylin and eosin staining of the samples for histopathological diagnosis and grading were determined by three staff pathologists using the World Health Organization criteria. All patients were screened for BRCA1 and BRCA2 mutations by multiplex polymerase chain reaction (PCR) with complete sequence analysis as previously described [11]; their characteristics are given in Additional file 1.

### Cell culture and lentiviral transfection

Primary ovarian cancer cells were obtained from the ascites of patients undergoing surgery for ovarian cancer and cultured in RPMI 1640 with 10% fetal bovine serum (Invitrogen, CA USA), using methods reported by Szlosarek [12]. Human 293 T cells and wild-type SKOV3 ovarian carcinoma cells were maintained in DMEM with 10% fetal bovine serum (Invitrogen). Lentiviral vectors expressing short hairpin RNAs (shRNAs) against BRCA1 (NM_007299) were obtained from GeneChem Co., Ltd (Shanghai, China), and synthesized as follows: Forward: 5′-CCGGAACCTGTCTCCACAAAGTGTGCTCGAGCACACTTTGT GGAGACAGGTTTTTTTG-3′, and Reverse: 5′-AATTCAAAAAAACCTGT CTCCACAAAGTGTGCTCGAGCACACTTTGTGGAGACAGGTT-3′. The non-silencing shRNA sequence was used as a negative control and synthesized as follows: forward, 5′-ccggTTCTCCGAACGTGTCACGTctcgagACGTGACACGTTCGGAGAAtttttg-3′, and reverse, 5′-aattcaaaaaTTCTCCGAACGTGTCACGTctcgagACGTGACACGTTCGGAGAA-3′. For overexpression of BRCA1, the open reading frame of BRCA1 (NM_007299) was cloned into the lentiviral vector GV287 (Ubi-MCS-3FLAG-SV40-EGFP; GeneChem Co., Ltd). Transfections were performed using polybrene and enhanced infection solution (GeneChem Co., Ltd) according to the manufacturer’s recommended protocol. The efficiency of BRCA1 knockdown and overexpression was shown in Additional file 2 (supplementary methods are shown in Additional file 3).

### Real-time quantitative PCR

Total RNA was extracted using Trizol reagents (Invitrogen) according to the manufacturer’s protocol. DNA contamination was removed by adding DNase I (Invitrogen) according to the manufacturer’s protocol. Total RNA was then reverse-transcribed from 2 μg of RNA using the PrimeScript RT Master Mix kit (TaKaRa, Dalian, China) and amplified by SYBR Premix Ex TaqTM II (TaKaRa) in a Roche LightCycler 2.0 instrument (Roche Diagnostics, Mannheim, Germany). The specific primer sequences were as follows: GR: 5′- TGTTTTGCTCCTGATCTGA -3′ (F) and 5′- TCGGGGAATTCAATACTCA-3′ (R); BRCA1: 5′-GGCTATCCTCTCAGAGTGACATTT-3′ (F) and 5′-GCTTTATCAGGTTATGTTGCATGG-3′ (R); GAPDH: 5′-AGGTGAAGGTCGGAGTCA-3′ (F) and 5′-GGTCATTGATGGCAACAA-3′(R). GAPDH mRNA was amplified as an internal control for normalization of each sample. All samples were analyzed in triplicate using the 2^–∆∆CT^ method.

### Immunohistochemistry

The standard SP kit (Zhongshan, Beijing, China) was used for immunohistochemical staining. Briefly, serial 4-μm sections were obtained from each paraffin-embedded tissue block. Following deparaffinization and rehydration, sections were subjected to microwave antigen retrieval. The primary antibodies were rabbit polyclonal anti-GR (1:100; Santa, Cruz Biotechnologies, USA) and rabbit polyclonal anti-BRCA1 (1:100; Santa), and the sections were incubated overnight at 4°C with this antibody. 3,3′-diaminobenzidine was used as the chromogen. Nuclei were counterstained with hematoxylin, and slides were dried and mounted. Negative controls were incubated with phosphate-buffered saline instead of the antibody. Immunostaining was evaluated by two independent pathologists, who were blinded to the identity of the subject groups. Area quantification was performed with a light microscope at a magnification of 400× and analyzed using Image-Pro Plus 6.0 (Media Cybernetics, USA). Intensity of the staining was scored by division into 10 arbitrary units based on the Mean Density: 0.00 to 0.05 = 0; 0.05 to 0.10 = 1; 0.10 to 0.15 = 2; 0.15 to 0.20 = 3; 0.20 to 0.25 = 4; 0.25 to 0.30 = 5; 0.30 to 0.35 = 6; 0.35 to 0.40 = 7; 0.40 to 0.45 = 8; >0.45 = 9. Any boundary values were classified as upper class.

### Bisulfite sequencing for BRCA1 promoter

All the tissues were used for bisulfite sequencing from the non-BRCA1-mutated cases. Genomic DNA extracted from ovarian cancer and normal ovarian tissue with a TIANamp Genomic DNA kit (Tiangen Biotech, Beijing, China) was subjected to bisulfite conversion using the EZ DNA Methylation-Direct kit (Zymo Research, Orange, USA) following the manufacturer’s instructions; the conversion efficiency was estimated to be at least 99.6%. It was then amplified by nested PCR. After gel purification, cloning and transformation into E. coli Competent Cells JM109 (TaKaRa), ten positive clones of each sample were sequenced to ascertain the methylation patterns of each CpG locus. The following primers were used for BRCA1 gene (Accession number: NG_005905;) promoter: round I, F: 5′-TTGTAGTTTTTTTAAAGAGT-3′ and R: 5′-TACTACCTTTACCCAAAACAAAA-3′; round II, F: 5′-GTAGTTTTTTTAAAGAGTTGTA-3′ and R: 5′-ACCTTTACCCAAAACAAAAA-3′. The conditions were as follows: 95°C for 2 min, 40 cycles of 30 s at 95°C, 30 s at 56°C and 45 s at 72°C, then 72°C for 7 min.

### Statistical analysis

Regression analysis was used to examine the possible relationship between GR and BRCA1 expression. The data are presented as means ± SD. Statistical differences in the data were evaluated by Student’s t-test or one-way ANOVA as appropriate, and were considered significant at *P* < 0.05.

## Results

### Differences in expression patterns of GR in non-mutated and BRCA1-mutated ovarian cancer

**Figure 1:**
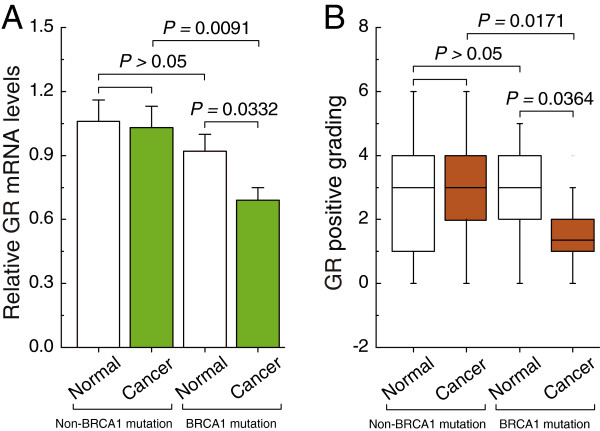
**GR expression patterns in non-mutated and BRCA1-mutated ovarian cancer. A**, relative GR mRNA levels were measured in 28 pairs of non-mutated and BRCA1-mutated ovarian cancer and their adjacent normal tissue. Bar graphs show mean ± SD. **B**, GR protein levels assessed by immunohistochemistry in 28 pairs of non-mutated and BRCA1-mutated ovarian cancer and their adjacent normal tissue. Intensity of the staining was scored by division into 10 arbitrary units based on the Mean Density (see details in Methods: Immunohistochemistry).

Real-time PCR and immunohistochemical analysis showed that there were no significant differences in the expression of GR mRNA and protein between non-BRCA1-mutated ovarian cancer and adjacent normal tissue (Figure 1A and B). It is, however, interesting to note that BRCA1-mutated ovarian cancer tissue showed dramatically reduced the expression of GR compared to adjacent normal tissue (Figure 1A and B). In contrast, expression levels of GR were not affected by BRCA2 mutations (Additional file 4).

### Hypermethylated BRCA1 promoter-mediated decreased expression of BRCA1 is positively correlated with GR levels

**Figure 2:**
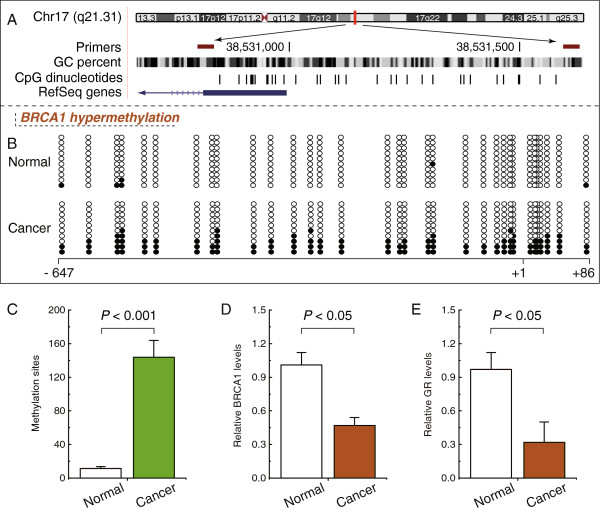
**GR expression patterns in ovarian cancer with hypermethylated promoter-mediated BRCA1 inactivation. A**, the location of CpG sites in the core promoter region of the BRCA1. Genomic coordinates are shown, along with the primer-amplified fragments, GC percentage, location of individual CpG dinucleotides (dashes), and BRCA1 RefSeq gene (exon 1 is shown as a blue box and the intron is shown as an arrowed line). The arrow indicates the direction of transcription. **B**, comparative analysis of methylation patterns in the core promoter region of BRCA1 in ovarian cancer and adjacent normal tissue. The circles correspond to the CpG sites denoted by black dashes in Figure 2A. Closed circles, methylation; open circles, unmethylated. Ten individual clones were sequenced for each sample. **C**, summary of the methylation levels of BRCA1 core promoter from the measurements shown in Figure 2B. **D**, relative BRCA1 mRNA levels were measured in ovarian cancer with identified hypermethylated BRCA1 promoter, compared with their adjacent normal tissue. **E**, relative GR mRNA levels were measured in ovarian cancer with identified BRCA1 inactivation, compared with their adjacent normal tissue. Each group, n = 22. Bar graphs show mean ± SD.

In mammals, promoter methylation at CpG dinucleotides is an important feature regulating gene expression [13]. Consistent with this idea, we showed that ovarian cancer tissue with a hypermethylated BRCA1 promoter (Figure 2B and C) displayed decreased expression of BRCA1 in comparison with adjacent normal tissue (Figure 2D); Figure 2A shows the location of CpG sites in BRCA1 promoter. Based on these considerations, the low levels of BRCA1 appeared to be mediated by promoter hypermethylation, making this an appropriate model to investigate the physiological relationship between BRCA1 and GR. Notably, the expression levels of GR decreased markedly (Figure 2E), along with hypermethylated promoter-mediated BRCA1 deficiency in ovarian cancer.

### BRCA1 is positively correlated with GR expression in non-BRCA1-mutated ovarian cancer samples

**Figure 3:**
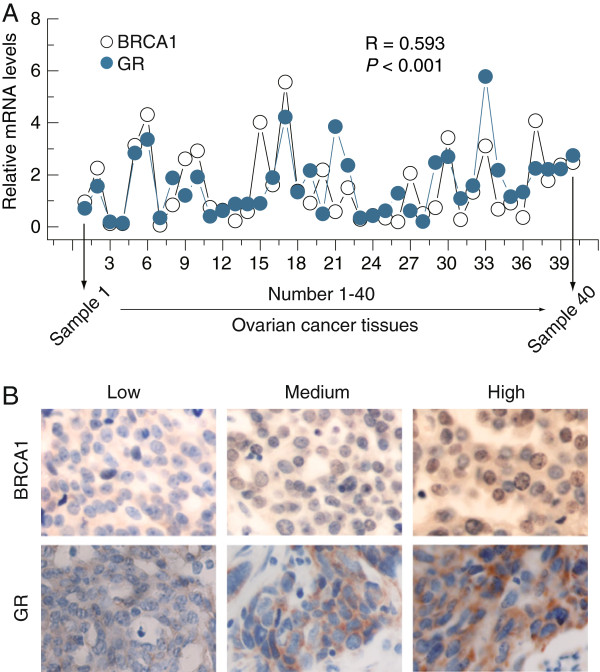
**Correlation between the expression levels of BRCA1 and GR in non-BRCA1-mutated ovarian cancer samples. A**, correlation between the BRCA1 and GR mRNA levels in 40 non-BRCA1-mutated ovarian cancer tissues. **B**, examples of immunohistochemical staining showing the positive correlation between the expression levels of BRCA1 and GR in 40 non-BRCA1-mutated ovarian cancer tissues. Magnification is 400×.

Of particular interest and potential clinical relevance, the relationship between BRCA1 and GR expression was studied in 40 non-BRCA1-mutated ovarian cancer specimens. Our results showed that there was a significant positive association between BRCA1 and GR protein expression (Figure 3A and B). Notably, as shown in Figure 3B, increased GR levels were mainly located in the cytoplasm, along with the increased expression of BRCA1 in ovarian cancer tissues.

### BRCA1 can regulate GR expression in ovarian cancer cells

**Figure 4:**
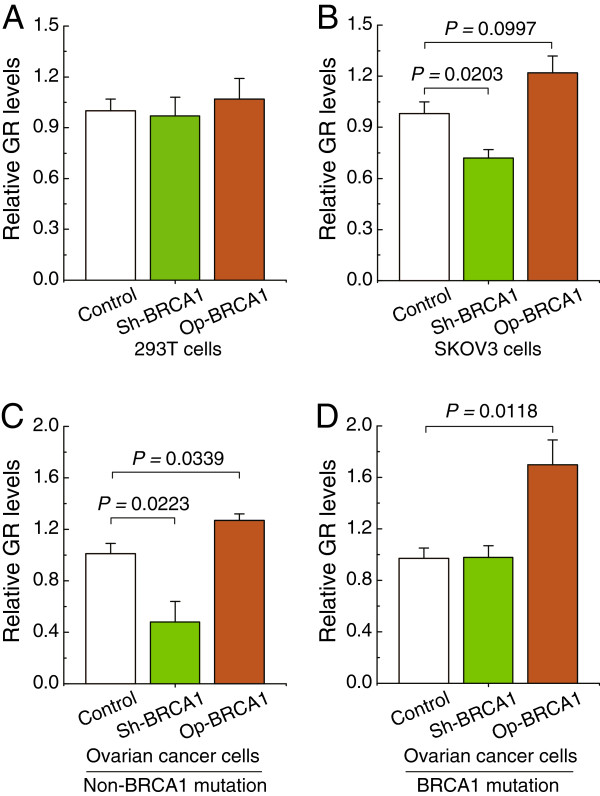
**Effects of BRCA1 on GR expression. A–D**, relative GR mRNA levels after the overexpression or knockdown of BRCA1 in 293 T cells, wild-type SKOV3 ovarian cancer cells, and primary non-mutated and BRCA1-mutated ovarian cancer cells (repeated 12 times for each group). Bar graphs show mean ± SD. Sh, short hairpin RNAs; Op, overexpression.

To confirm the role of BRCA1 in the regulation of GR, the effects of overexpression or knockdown of BRCA1 were observed in 293 T cells, the human ovarian carcinoma cell line SKOV3, and primary ovarian cancer cells with identified BRCA1 mutations or those which were non-mutated. The results indicated that there were no significant changes in the expression of GR after overexpression or knockdown of BRCA1 in 293 T cells (Figure 4A). Interestingly, we observed that overexpression of BRCA1 was an effective way to induce an increase in GR levels in SKOV3 cells, primary non-mutated and BRCA1-mutated ovarian cancer cells (Figure 4B-D). BRCA1 knockdown effectively inhibited the expression of GR in SKOV3 cells and primary non-BRCA1-mutated ovarian cancer cells (Figure 4B and C). GR levels were not sensitive to the BRCA1 knockdown in primary BRCA1-mutated ovarian cancer cells (Figure 4D).

## Discussion

In this study, we report for the first time an association between BRCA1 and GR status in ovarian cancer: (i) the BRCA1 inactivation group showed dramatically decreased expression of GR compared with adjacent normal tissue; (ii) there was a positive correlation between BRCA1 and GR expression in human ovarian cancer specimens; (iii) BRCA1 knockdown was effective at inhibiting GR expression, and overexpression of BRCA1 induces an increase in GR levels in ovarian cancer cells. These results suggest that GR may be a potential target for BRCA1 in ovarian cancer. However, the regulatory effects of BRCA1 on GR were only observed in ovarian cancer cells; 293 T cells were insensitive to the knockdown or overexpression of BRCA1. In addition, it is noteworthy that increased GR levels were mainly located in the cytoplasm nor the nuclei, along with the increased expression of BRCA1. Therefore, it appears that BRCA1 may be involved in the inhibition of GR transfer to the nucleus, which is consistent with previous findings [9]. Notably, a growing body of data suggests that there is extensive crosstalk among BRCA1 signaling pathways and several hormone receptors. For example, both the insulin-like growth factor 1 receptor [3] and epidermal growth factor receptor [14] are downstream targets for BRCA1; BRCA1 inhibits the transcriptional activity of progesterone receptor (PR) in the PR-positive breast cancer cell line T47D [15]; and ER-alpha activity can be suppressed by BRCA1 through regulating the acetylation *vs*. ubiquitination [16]. However, there have been few reports about the interactions between BRCA1 and GR in ovarian cancer. It is interesting to note that loss of function of the tumor suppressor gene BRCA1 plays an important role in promoting cell proliferation and survival [17,18]. The mechanism may involve: 1) inducing insulin-like growth factor 1 expression [3,19] in an estrogen receptor α-dependent manner [19,20], and 2) stimulating PR activity by facilitating progesterone binding to the progesterone response elements [21]. Remarkably, GR has an anti-proliferative effect on various cells [22]. Therefore, the discovery of BRCA1- inactivation mediated GR repression will stimulate new interest in BRCA1-related cellular proliferation. To date, it is not fully understood how BRCA1 activates GR transcription at the molecular level. However, some insight was gained by a recent study which reported that BRCA1 can regulate GR activity through GR phosphorylation on Ser211 by modulating MAPK p38 in MCF-7 and MDA-MB-231 cell lines [9].

## Conclusions

Our results indicate that BRCA1 may be a potential regulator of GR in ovarian cancer cells. Based on these findings, there are some interesting issues that need to be considered in future studies; for example, how BRCA1 affects GR transcription and whether other factors could cooperate with BRCA1 in controlling GR expression. Also, the complex interactions between BRCA1 and GR signaling pathways need to be clarified. All of this may improve our understanding of the basic molecular mechanism of BRCA1-related ovarian cancer.

## Abbreviations

ANOVA: Analysis of variance; GR: Glucocorticoid receptor; PCR: Polymerase chain reaction; PR: Progesterone receptor; shRNAs: short hairpin RNAs.

## Competing interests

The authors declare that they have no competing interests.

## Authors’ contributions

DL conceived of the study, participated in its design and drafted the manuscript. DL, YYF and CC carried out data acquisition and interpretation. YYF, CYL and TTL participated in the design of the study and performed the statistical analysis. All authors read and approved the final manuscript.

## Pre-publication history

The pre-publication history for this paper can be accessed here:

http://www.biomedcentral.com/1471-2407/14/188/prepub

## Supplementary Material

Additional file 1: Table S1 Clinical characteristics for the 28 BRCA1-mutated serous ovarian cancer patients. **Table S2.** Clinical characteristics for the 23 BRCA1-mutated serous ovarian cancer patients. Click here for file

Additional file 2 BRCA1-knockdown efficiency. Click here for file

Additional file 3 Supplementary Methods. Click here for file

Additional file 4 GR expression patterns in non-mutated and BRCA2-mutated ovarian cancer. Click here for file
