# Supplementary material for: Glucocorticoid receptor repression mediated by BRCA1 inactivation in ovarian cancer
Source: BMC Cancer. 2014 Mar 14;14:188. doi: 10.1186/1471-2407-14-188 (PMC4004164; doi:10.1186/1471-2407-14-188)
Supplement: Additional file 1: Table S1 — Clinical characteristics for the 28 BRCA1-mutated serous ovarian cancer patients. Table S2. Clinical characteristics for the 23 BRCA1-mutated serous ovarian cancer patients. [file 1471-2407-14-188-S1.pdf]

Table S1 Clinical characteristics for the 28 BRCA1-mutated serous ovarian cancer patients

Case	Age	Stage[a]	Grade[b]	Gene	Exon	Mutation	AA change	Mutation type[c]
1	70	IIIC	PD	BRCA1	11	c.2331 T > A	p.Y777X	NS
2	47	IIIC	MD	BRCA1	11	c.2311 T > G	p.L771V	MS
3	54	IIIC	PD	BRCA1	11	c.2566 T > C	p.Y856H	MS
4	68	IIIC	MD	BRCA1	11	c.2612 C > T	p.P871L	MS
5	64	IIIC	MD	BRCA1	11	c.2612 C > T	p.P871L	MS
6	57	IA	PD	BRCA1	11	c.2566 T > C	p.Y856H	MS
7	63	IIIC	PD	BRCA1	11	c.2612 C > T	p.P871L	MS
8	56	IIIC	PD	BRCA1	11	c.2709 T > A	p.C903X	NS
9	49	IIB	PD	BRCA1	11	c.3710 C > T	p.A1237V	MS
10	52	IIB	MD	BRCA1	11	c.3109 C > T	p.Q1037X	NS
11	58	IIIC	PD	BRCA1	11	c.2212 G > A	p.V738I	MS
12	61	IIIC	PD	BRCA1	11	c.2312 T > C	p.L771S	MS
13	53	IIIC	PD	BRCA1	11	c.2363 T > C	p.V788A	MS
14	57	IIIC	PD	BRCA1	11	c.2429 A > G	p.N810S	MS
15	66	IIIC	MD	BRCA1	11	c.2741 A > G	p.E914G	MS
16	58	IIB	MD	BRCA1	8	c.470-471delCT	p.S157X	NS
17	70	IA	MD	BRCA1	8	c.491 C > T	p.T164I	MS
18	48	IIIC	PD	BRCA1	2	c.63 C > G	p.I21M	MS
19	51	IA	PD	BRCA1	11	c.1010delA	p.K339RfsX2	FS
20	50	IIB	MD	BRCA1	3	c.127 T > A	p.F43I	MS
21	62	IIIC	PD	BRCA1	11	c.4046_4047insG	p.E1352GfsX4	FS
22	49	IIIC	PD	BRCA1	14	c.4372 C > T	p.Q1458X	NS
23	55	IIIC	MD	BRCA1	14	c.4454 C > T	p.T1485I	MS
24	64	IIB	PD	BRCA1	24	c.5666 G > C	p.G1889A	MS
25	52	IIB	PD	BRCA1	24	c.5906 T > C	p.F1969S	MS
26	68	IIIC	PD	BRCA1	24	c.6489 T > A	p.F2163L	MS
27	61	IIIC	MD	BRCA1	24	c.6677C > G	p.S2226X	NS
28	58	IIB	PD	BRCA1	24	c.6856 G > T	p.D2286Y	MS

Table S2 Clinical characteristics for the 23 BRCA2-mutated serous ovarian cancer patients

Case	Age	Stage[a]	Grade[b]	Gene	Exon	Mutation	AA change	Mutation type[c]
1	51	IIIC	PD	BRCA2	3	c.147 A > T	p.E49D	MS
2	58	IIB	PD	BRCA2	4	c.380 C > T	p.A127V	MS
3	69	IIIC	PD	BRCA2	11	c.3109 C > T	p.Q1037X	NS
4	48	IIIC	MD	BRCA2	10	c.945 T > A	p.C315X	NS
5	56	IA	MD	BRCA2	10	c.1022 G > C	p.C341S	MS
6	63	IIB	PD	BRCA2	10	c.1062 del T	p.F354LfsX4	FS
7	70	IIIC	PD	BRCA2	10	c.1169C > T	p.S390F	MS
8	62	IIIC	PD	BRCA2	10	c.1261C > T	p.Q421X	NS
9	55	IIB	MD	BRCA2	10	c.1652 A > T	p.D551V	MS
10	49	IIIC	PD	BRCA2	11	c.3109 C > T	p.Q1037X	NS
11	68	IIIC	PD	BRCA2	10	c.1062 del T	p.F354LfsX4	FS
12	65	IIIC	PD	BRCA2	11	c.5164-5165delAG	p.S1722YfsX4	FS
13	59	IIIC	MD	BRCA2	11	c.2981 C > T	p.A994V	MS
14	66	IIB	PD	BRCA2	11	c.3410 T > A	p.L1137X	NS
15	54	IIIC	PD	BRCA2	11	c.2981 C > T	p.A994V	MS
16	51	IA	MD	BRCA2	11	c.3710 C > T	p.A1237V	MS
17	49	IIIC	PD	BRCA2	11	c.5722-5723delCT	p.L1908RfsX2	FS
18	50	IIIC	MD	BRCA2	11	c.3710 C > T	p.A1237V	MS
19	67	IIB	PD	BRCA2	11	c.6539 T > A	p.L2180X	NS
20	62	IIIC	PD	BRCA2	11	c.3109 C > T	p.Q1037X	NS
21	58	IIIC	PD	BRCA2	24	c.10115 C > A	p.A3372D	MS
22	70	IIIC	MD	BRCA2	24	c.10373 G > C	p.S3458T	MS
23	55	IIB	PD	BRCA2	24	c.10739 A > C	p.N3580T	MS

a: The tumor stages were assessed according to the International Federation of Gynecology and Obstetrics.

b: PD: poorly differentiated; MD: moderately differentiated.

c: NS: nonsense mutation; MS: missense mutation ; FS: frame-shift.
