# Supplementary material for: Glucocorticoid receptor repression mediated by BRCA1 inactivation in ovarian cancer
Source: BMC Cancer. 2014 Mar 14;14:188. doi: 10.1186/1471-2407-14-188 (PMC4004164; doi:10.1186/1471-2407-14-188)
Supplement: Additional file 2 — BRCA1-knockdown efficiency. [file 1471-2407-14-188-S2.pdf]

Additional file 2 - BRCA1-knockdown efficiency

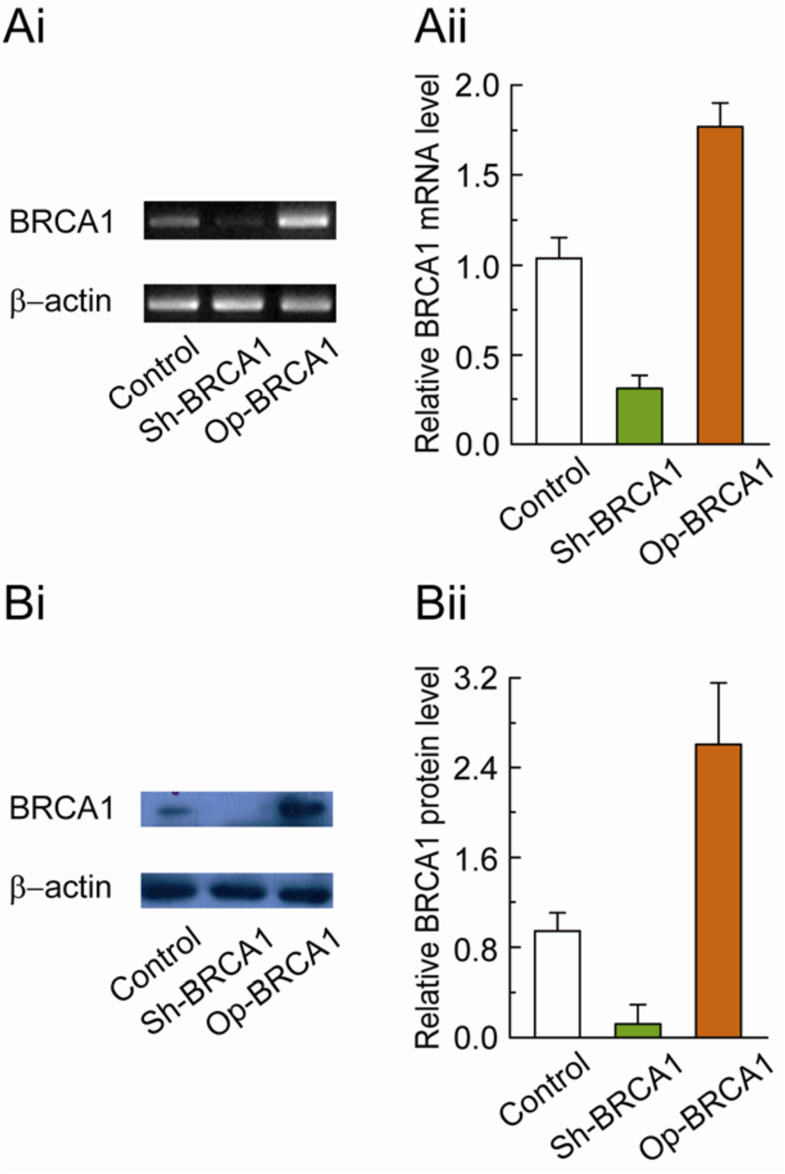

Semi-quantitative PCR (Ai) and western blotting (Bi) showing BRCA1 levels before, and after knockdown or overexpression of BRCA1. Aii and Bii, the results from three independent experiments. Bar graphs show mean ± SD. Sh, short hairpin RNAs; Op, overexpression.
