# Supplementary material for: Glucocorticoid receptor repression mediated by BRCA1 inactivation in ovarian cancer
Source: BMC Cancer. 2014 Mar 14;14:188. doi: 10.1186/1471-2407-14-188 (PMC4004164; doi:10.1186/1471-2407-14-188)
Supplement: Additional file 3 — Supplementary Methods. [file 1471-2407-14-188-S3.pdf]

Additional file 3 - Supplementary Methods

Semi-quantitative PCR

BRCA1-knockdown efficiency was measured by semi-quantitative PCR. Detailed isolation and reverse-transcribed protocols were established as described in the text (Methods). The specific primer sequences were purchased from Santa Cruz Biotechnology (CA, USA): sc-29219-PR. PCR amplification was performed in a Techne TC-512 gradient thermal cycler (Progene, Techne Ltd., Cambridge, UK). PCR reaction conditions were as follows: 95 °C for 10 min; 32 cycles of 95 °C for 30 s, 60 °C for 30 s and 72 °C for 45 s; followed by an extension reaction at 72 °C for 10 min. The reaction products were analyzed by agarose gel electrophoresis and visualized by UV light after staining with ethidium bromide. The similar results were obtained in primary ovarian cancer cells, 293T cells and SKOV3 ovarian cancer cells. sh: short hairpin RNAs; op: overexpression.

Western blotting

BRCA1-knockdown efficiency was assayed by western blotting. Western blotting analysis of BRCA1 was performed at 48 h after transfection according to standard protocols. The protein concentration was determined by the Bio-Rad Protein Assay Kit (Bio-Rad, Hercules, CA, USA). Briefly, 30 μg protein was separated by 8% SDS polyacrylamide gels, and transferred to polyvinyl difluoride membranes (Millipore, MA, USA). The membranes were blocked in TBS containing 0.1% Tween-20 and 5% non-fat dry milk for 60 min at room temperature, and incubated with antibody to BRCA1 (sc-642) (1:500; Santa Cruz Biotechnology, CA, USA) overnight at 4 °C. Then, the membranes were washed by PBS-Tween followed by 1 h incubation at room temperature with horseradish peroxidase-conjugated secondary antibody (1:5000; Santa Cruz Biotechnology, CA, USA) and detected using the enhanced chemiluminescence (Amersham Life Science, NJ, USA). The similar results were obtained in primary ovarian cancer cells, 293T cells and SKOV3 ovarian cancer cells. sh: short hairpin RNAs; op: overexpression.
