# Supplementary material for: Glucocorticoid receptor repression mediated by BRCA1 inactivation in ovarian cancer
Source: BMC Cancer. 2014 Mar 14;14:188. doi: 10.1186/1471-2407-14-188 (PMC4004164; doi:10.1186/1471-2407-14-188)
Supplement: Additional file 4 — GR expression patterns in non-mutated and BRCA2-mutated ovarian cancer. [file 1471-2407-14-188-S4.pdf]

Additional file 4 - GR expression patterns in non-mutated and BRCA2-mutated ovarian cancer

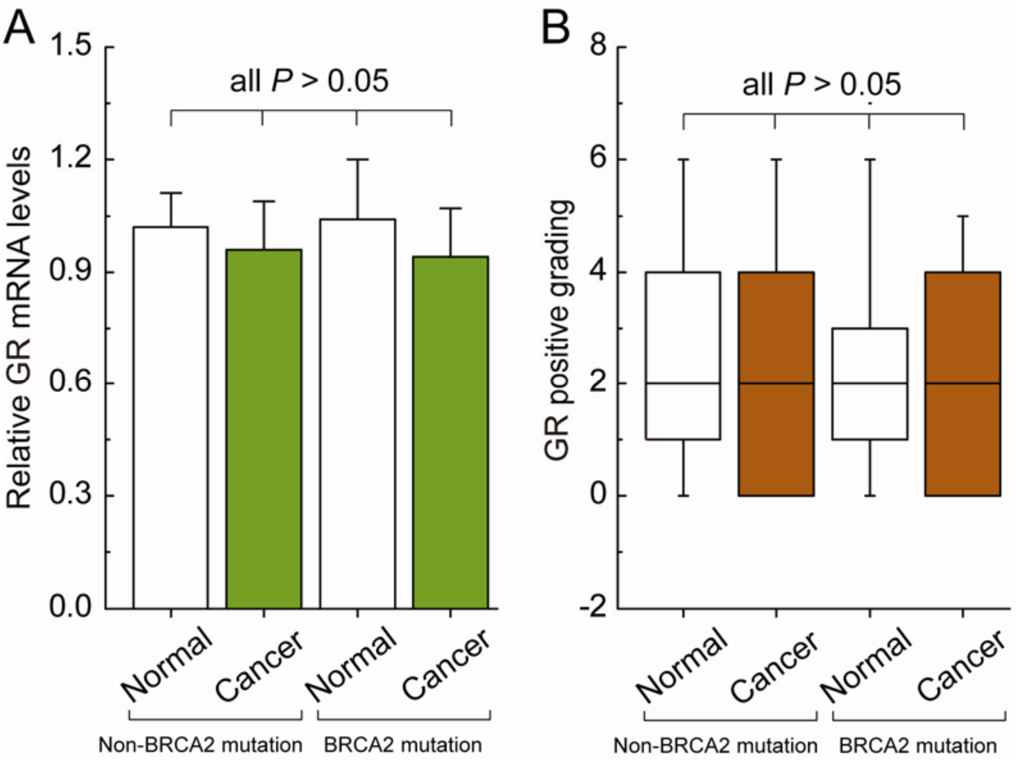

A, relative GR mRNA levels were measured in 23 pairs of non-mutated and BRCA2-mutated ovarian cancer, and their adjacent normal tissue. Bar graphs show mean ±SD. B, GR protein levels assessed by immunohistochemistry in 23 pairs of non-mutated and BRCA2-mutated ovarian cancer, and their adjacent normal tissue. The intensity of staining was divided into 10 units.
